# Coexisting CLT in PTC is an independent predictor of tumor aggressiveness for patients aged under 55: a retrospective analysis of 635 patients

**DOI:** 10.1186/s12902-022-00945-4

**Published:** 2022-03-07

**Authors:** Bing’e Ma, Xiyi Chen, Zhengping Zhao, Xiaoyang Yin, Qin Ji, Yifan Zhou, Chaoqun Ma, Jianhua Wang

**Affiliations:** 1 Department of Thyroid and Breast Surgery, Affiliated Hospital of Integrated Traditional Chinese and Western Medicine, Nanjing University of Chinese Medicine, Nanjing, 210028 China; 2 The Fourth School of Clinical Medicine, Nanjing Medical University, Nanjing, 210029 China; 3 The First School of Clinical Medicine, Nanjing University of Chinese Medicine, Nanjing, 210023 China; 4 Department of General Surgery, Affiliated Hospital of Nanjing University of Chinese Medicine, Nanjing, 210029 China; 5 Jiangsu Province Academy of Traditional Chinese Medicine, Nanjing, 210028 China

**Keywords:** Papillary thyroid cancer, Chronic lymphocytic thyroiditis, Extrathyroidal extension, Recurrence risks, Age stratification

## Abstract

**Background:**

The study was aimed at investigating the potential role of chronic lymphocytic thyroiditis (CLT) in papillary thyroid cancer (PTC) aggressiveness for patients aged below 55, as well as to figure out factors influencing potential recurrence risk in different age groups.

**Methods:**

A total of 635 adult patients were retrospectively analyzed. 188 patients were diagnosed with coexistent CLT and the remaining 447 were classified as non-CLT. Then the characteristics of CLT-coexisted patients and non-CLT ones were compared respectively when patients were aged ≥ 55 years or below. The association among postoperative clinicopathological features were also analyzed using multivariate regression. In addition, the prognostic value of several variables relating to high-risk recurrence were estimated within different age groups.

**Results:**

When divided in two age groups (55 years as the borderline), non-CLT group (aged below 55 years) had a remarkable frequency of small size lesion (D_max_ ≤ 1 cm) compared with CLT-coexisted patients (54.6% to 43.0%, *p* = 0.02). In addition, non-CLT patients tended to have intrathyroidal extension as opposed to those with coexistent CLT (20.2% to 28.2%, *p* = 0.05). In multivariate analysis, CLT still significantly acted as an independent risk factor of greater lesion size (D_min_ > 1 cm) (OR = 1.7, *p* = 0.02) and mildly promoted gross extrathyroidal extension (ETE) (OR = 1.4, *p* = 0.06). However, associations didn’t emerge in the characteristics mentioned above with CLT when patients were ≥ 55 years old. The prognostic value of CLT in high-risk recurrence was evident only in patients aged 35–44 years. (OR = 2.4, 95%CI:1.2–5.4, *p* = 0.02). Greater lesion size independently promoted gross ETE, no matter patients were aged above 55 years or not. Its prognostic value of high-risk recurrence was significant throughout all age groups.

**Conclusion:**

These findings revealed that CLT coexistence might be the unfavorable factor of PTC aggressiveness in patients aged below 55 years. Its role as well as greater tumor size may potentially predict higher recurrence risk according to results figured out in the prediction model.

**Supplementary Information:**

The online version contains supplementary material available at 10.1186/s12902-022-00945-4.

## Background

The most common histologic type of thyroid malignancy is papillary thyroid cancer (PTC), which accounts for about 90% of all thyroid malignant carcinomas [1]. It has been reported more frequently than ever due to the accuracy and convenience of diagnostic technology such as ultrasound and fine-needle aspiration, which leads mainly to the increase of overall thyroid cancer incidence [2]. Moreover, analyses from three SEER cohorts (SEER-9, SEER-13 and SEER-18) all indicate the significant increase in age-adjusted incidence-based mortality rates over their respective subset of registry year to 2017 [3]. Poor prognosis still remains with high-risk features including extrathyroidal extension and evident metastasis, which necessitate total thyroidectomy [4, 5].

Chronic lymphocytic thyroiditis (CLT) is an autoimmune disease histopathologically characterized by diffused infiltration of autoreactive T and B cells (diffused lymphocyte infiltration, CLT) [6]. Since it was first described by Dailey et al. [7] in 1955, a large amount of studies have carried out the controversial relationship between PTC and CLT [8–10]. Considering the subclinical course in both disease and incidental diagnosis sometimes, conflicting data and conclusions are undoubtedly raised. It has been reported that CLT serves as an important role in the development of PTC [11] and increases the risk of PTC incidence [12, 13]. Of note however, there still exists the advocation that CLT is associated with less aggressive clinicopathological presentation and a better prognosis of PTC [14–16]. The molecular link between CLT and PTC should be noted. As the most frequent genetic alteration occurring in PTC, BRAF^V600E^ has long been regarded as the independent negative factor of PTC outcome[17, 18]. Recent reports have indicated that aggression of PTC with BRAF^V600E^ positivity was alleviated when CLT was present, including smaller lesion size and less extracapsular extension[19]. In contrast, some reports also argued that CLT has no effect, or even negative effect, on the outcome of PTC [20, 21].

Besides, staging system for assessing PTC recurrence, which is one of the major concerns regarding treatments, has raised great attention[22, 23]. Specifically, in terms of the association between CLT and PTC recurrence, many studies have suggested the strong link of coexistent CLT with a lower risk of recurrence [14, 16, 24–26]. However, the definition of “recurrence” in some of these studies actually referred to “persistence” because former “disease free” condition was not confirmed (usually refers to the absence of thyroglobulin or antithyroid globulin antibodies [27–29]). A recurrence is defined as new biochemical (suppressed Tg ≥ 1 ng/ml, and/or stimulated Tg ≥ 2 ng/ml), functional imaging evidence (18-FDG-PET scan or RAI scan) or positive scan imaging of tumors (structural recurrence) detected after disease-free conditions [27–29]. In that case, a predicting model of different recurrence risks was in need. According to the 2015 American Thyroid Association (ATA) Management Guidelines, three-tiered stratification system of structural recurrence risks was highly recommended for PTC patients after initial thyroidectomy, which was based on presence of several clinicopathologic features, including gross extrathyroidal extension and numbers of involved lymph nodes [30]. These characteristics have also been observed due to the need to ensure their associations with coexistent CLT in PTC patients.

Herein, combined with the background mentioned above, our retrospective study was conducted to analyze the differences in clinicopathologic characteristics between adult PTC patients (≥ 18 years) with contrary CLT status. Our main objective is to investigate the potentially negative role of CLT in tumor aggressiveness of patients aged below 55 years, as well as in estimating high-risk recurrence related factors in different age groups.

## Methods

### Eligibility criteria and patient background

The retrospective analysis starts in December 2019, which collects 635 adult patients diagnosed with papillary thyroid cancer between February 2014 and December 2017 from all those 654 people confirmed as thyroid cancers, no matter what types, by histopathological examinations in the pathology department of Jiangsu Province Hospital on Integration of Chinese and Western Medicine. For the whole studied group, only patients diagnosed with PTC (*n* = 635) were enrolled, leaving other types excluded. All clinical features, including age, sex, preoperative ultrasound results and pathologic examinations were reviewed and confirmed according to previous electronic records. All patients underwent thyroidectomy or lobectomy for the first time.

Patients suspicious for bilateral malignancy in preoperative fine-needle aspiration biopsy and lateral lymph nodes metastases according to preoperative imaging evaluation all underwent total thyroidectomy and lateral neck dissections. Besides, those with lesions sized above 4 cm at maximum diameter, found in extrathyroidal extension, invading recurrent laryngeal nerves or extrathyroidal muscles, which were indicated in frozen sections, were operated total thyroidectomy as well. Patients in absence of any features mentioned above underwent lobectomy otherwise. All the operations were excellently accomplished by an experienced surgeon who has been engaged in thyroid surgery for over two decades, and the postoperative biopsy was examined and reviewed by two seasoned pathologists.

### Histopathological examinations

CLT was histopathologically defined as the presence of mononuclear lymphocytes infiltration in thyroid parenchyma and stroma, as well as a few reactive germinal centers with lymphoid follicle formation, parenchymal atrophy and abundant oxyphilic cell changes in follicular cells [31–33]. Multifocality means that at least one lobe was discovered with more than one lesion existing. Lesion size was initially divided as maximum diameter ≤ 1 cm or minimum lesion diameter above 1 cm. In the lesion size rank comparison and final analysis of recurrence risk stratification, lesions sized > 1 cm were subdivided further in 3 groups, which were 1 cm < D_max_ ≤ 2 cm, 2 cm < D_max_ ≤ 4 cm and D_max_ > 4 cm in accordance with the “T” categorization in 8^th^ edition of TNM staging.

### Postoperative stratification

Assessment of each patient’s clinical stage was according to the 8^th^ edition of TNM system (2016), for which the histopathology associated classification is re-estimated on the basis of pathology reports. In addition, MACIS score is used for differentiated thyroid cancer, and the calculation was based on several indexes, including age, tumor size, whether with resecting operation, local invasiveness or distant metastasis [34]. Sex, age, preoperative CLT, as well as pathologic evidence of tumor size, central lymph node metastasis (CLNM), lateral lymph node metastasis (LLNM), multifocality, TNM staging and gross extrathyroidal extension after surgery were recorded for each patient involved. Gross extrathyroidal extension (ETE) refers to the gross soft-tissue invasion identified on clinical examination, intraoperatively or on imaging, which conveys an increased risk of mortality. The newly modified clinicopathologic recurrence risk stratification system was proposed in the 2015 version of the American Thyroid Association (ATA) thyroid cancer guidelines, and it was used to classify patients as low-, intermediate- or high-risk recurrence [30]. In our study, patients confirmed with gross ETE were all classified as having high-risk recurrence. Those with absence of gross ETE but diagnosed with > 5 involved pathologic LN metastasis, of which the largest dimension was < 3 cm, were classified in intermediate-risk recurrence. Otherwise, the patients would be classified in low-risk recurrent disease, who were characterized with no local or distant metastases, and with absence of locoregional invasion. Patients were divided into five groups by age (10 years as a span when below 55 years old), so our five age groups were stratified as 18–24, 25–34, 35–44, 45–54 and ≥ 55 years, which was applied to analyzing the role of CLT predicting risks of recurrence.

### Statistical analysis

**Fig. 1:**
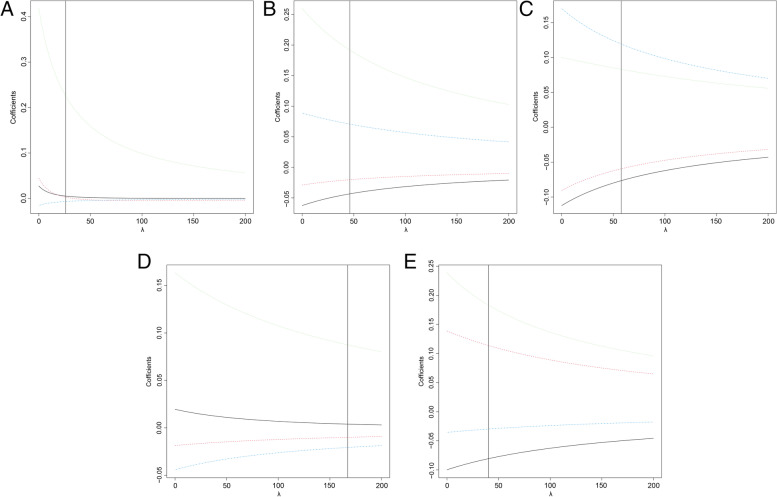
Ridge trace in different age groups. **A**:18–24 years. **B**:25–34 years. **C**:35–44 years. **D**:45–54 years. **E**: 55–75 years

The final recurrence risk stratification analysis (Table 7 and Fig. 1) was realized in R Windows version 3.6.3, and all the other data processing was undertaken with IBM SPSS Statistics for MacOS version 23.0.

The Pearson’s Chi Square test and contingency tables were used to compare categorical variables. The comparisons between non-normally distributed variables ordinally reorganized (including mean lesion size of non-CLT and CLT-coexistent groups) were completed by Mann–Whitney U test. Otherwise, the independent t-test would be utilized for distributed ones. Binary logistic regression analysis was applied to estimate odds ratio (OR) for dichotomous dependent variables. For ordered dependent variables, ordinal regression analysis was in use. Besides, we employed the collinearity diagnostics to exclude collinearity problems. Results showed that variance inflation factor (VIF) was far below 10 and tolerance was higher than 0.1 between variables in the multiple regression model. No collinearity existed among sex, age, CLT and relevant dependent variables in logistic regression. Ridge regression was applied because collinearity occurred when adding lesion size subdivision as the independent factor. The “lm.ridge” in MASS package was selected to produce GCV and coefficients, while the “linearRidge” in ridge package reports coefficients and *P* values. *P* value < 0.05 was considered statistically significant.

## Results

### Basic Clinicopathological features in all patients

Among the 635 PTC patients evaluated in the study, 188 (29.6%) had coexisting CLT while the remaining 447 (70.1%) were found to be non-CLT. The proportion of female in non-CLT and CLT-coexisted group was 70.5% and 89.9% (*p* < 0.001), with mean ages being 43.6 ± 11.9 and 44.1 ± 12.3 (*p* = 0.65) for each. A total of 325 patients were diagnosed as small lesion (with largest lesion diameter ≤ 1 cm) and 143 patients were confirmed with gross extrathyroidal extension (ETE). 623 patients were classified in TNM stage I, leaving only 12 patients in stage II-IV, which indicates that most patients enrolled were in early stage at diagnosis (Table 1).

**Table 1:** Clinicopathological characteristics of non-CLT and CLT-coexisted groups in all patients involved

Characteristics	Total	non-CLT(%)	CLT-coexisted(%)	OR(95%CI)	*P* value
Number of patients	635	447 (70.1)	188 (29.6)		
Sex					
Female	484	315 (70.5)	169 (89.9)	**3.7 (2.2–6.2)**	** < 0.001**
Male	151	132 (29.5)	19 (10.1)		
Age					
< 55	515	366 (71.1)	149 (28.9)	1.1 (0.8–1.6)	0.49
≥ 55	120	81 (67.5)	39 (32.5)		
^#^Mean rank of lesion size		307.67	342.55		**0.017**
0 < size ≤ 1 cm		240 (53.7)	85 (45.2)		
1 cm < size ≤ 2 cm		95 (21.3)	38 (20.2)		
2 cm < size ≤ 4 cm		108 (24.2)	61 (32.4)		
4 cm < size		4 (0.9)	4 (2.1)		
Small lesion (Dmax ≤ 1 cm)	325	240 (53.7)	85 (45.2)	**1.4 (1.0–2.0)**	**0.05**
Extrathyroidal extension	143	94 (21.0)	49 (26.1)	1.3 (0.9–2.0)	0.17
CLNM	297	219 (49.0)	78 (41.5)	0.7 (0.5–1.0)	0.08
LLNM	54	40 (8.9)	14 (7.4)	0.8 (0.4–1.5)	0.54
CLNM of small lesions	145	119(40.3)	26 (24.3)	0.5 (0.3–0.8)	**0.003**
TNM stage					
I	623	438 (98.0)	185 (98.4)	0.8 (0.2–2.9)	0.72
II-IV	12	9 (2.0)	3 (1.6)		
Multifocality	194	133 (29.8)	61 (33.0)	1.2 (0.8–1.7)	0.43
MACIS score					
< 6	595	417 (93.3)	178 (94.7)	1.3 (0.6–2.7)	0.51
6–6.99	31	24 (5.4)	7 (3.7)	0.7 (0.3–1.6)	0.38
7–7.99	9	6 (1.3)	3 (1.6)	1.2 (0.3–4.8)	0.81
High-risk recurrence	143	94 (21.0)	49 (26.1)	1.3 (0.9–2.0)	0.17

*CLNM* central lymph node metastasis, *LLNM* lateral lymph node metastasis. *OR* odds ratio, ^#^Mann–Whitney U test used

### CLT and features of PTC

With regard to the presence of extrathyroidal extension, there were 49 cases in CLT-coexisted group and 94 (26.1% to 21.0%, *p* = 0.166) in the non-CLT one. Mean sizes of non-CLT and CLT-coexisted groups were compared in ordinal format and the latter one was associated with larger lesions overall (*p* = 0.017). The CLT-coexisted group seemed to have a smaller portion of primary lesion size ≤ 1 cm than the non-CLT coexisted group (45.2% to 53.7%, *p* = 0.05), and there was a trend to have central LN metastasis in patients without coexistent CLT, although the difference didn’t reach set significance (49.0% to 41.5%, *p* = 0.08). Among patients with “small lesions”, absence of CLT-coexistence seems to be associated with central LN metastasis (40.3% to 24.3%, *p* = 0.003) (Table 1), but no significance was suggested in the relationship between CLT and LLNM, multifocality or gross ETE (Supplementary Table). No evident differences were shown in high-risk recurrence stratification between two groups (*p* = 0.17). There was no statistically significant difference in multifocality, TNM staging or MACIS score (Table 1).

### Age and CLT in PTC patients

A total of 515 patients were aged < 55 years, of whom 149 (28.9%) were diagnosed with coexisting CLT and the other 366 (71.1%) without. CLT-coexisted group had more female patients than the CLT-coexisted one (89.3% to 68.3%, *p* < 0.001). Mean lesion size of CLT-coexistent group was significantly larger than the other group (*p* = 0.005). There seemed to be fewer patients diagnosed as small lesion in CLT-coexisted group compared with the non-CLT group (43.0% to 54.6%, *p* = 0.02). The gross ETE was found more prevalent in CLT-coexisted group than the non-CLT one (28.2% to 20.2%, *p* = 0.04). There wasn’t any significant difference shown in CLNM (55.2% to 47.7%, *p* = 0.12) or LLNM (10.7% to 9.4%, *p* = 0.67) presence between CLT-coexistent and non-CLT patients. However, when lesion size was limited below 1 cm, central LN metastasis was more prevalent in non-CLT group (47.3% to 29.4, *p* = 0.004) (Table 2).

**Table 2:** Association between CLT and clinicopathological characteristics in patients < 55 years

Characteristics	Total	non-CLT(%)	CLT-coexisted(%)	OR(95%CI)	*P* value
Number of patients	515	366 (71.1)	149 (28.9)		
Sex					
Female	383	250 (68.3)	133 (89.3)	**3.9 (2.2–6.8)**	** < 0.001**
Male	132	116 (31.7)	16 (10.7)		
^#^Mean rank of lesion size		247.31	284.25		**0.005**
0 < size ≤ 1 cm		240 (53.7)	85 (45.2)		
1 cm < size ≤ 2 cm		95 (21.3)	38 (20.2)		
2 cm < size ≤ 4 cm		108 (24.2)	61 (32.4)		
4 cm < size		4 (0.9)	4 (2.1)		
Small lesion (Dmax ≤ 1 cm)	264	200 (54.6)	64 (43.0)	**1.6 (1.2–2.4)**	**0.02**
Extrathyroidal extension	116	74 (20.2)	42 (28.2)	**1.5 (1.1–2.4)**	**0.04**
CLNM	273	202 (55.2)	71 (47.7)	0.7 (0.5–1.1)	0.12
LLNM	53	39 (10.7)	14 (9.4)	0.9 (0.5–1.7)	0.67
CLNM of small lesions	139	114 (47.3)	25 (29.4)	0.5 (0.3–0.8)	**0.004**
TNM stage					
I	515	366 (71.1)	149 (28.9)		
I-IV	0				
Multifocality	152	102 (27.9)	50 (34.0)	1.3 (0.9–2.0)	0.27

*CLNM* central lymph node metastasis, *LLNM* lateral lymph node metastasis, *OR* odds ratio, ^#^Mann–Whitney U test used

In all the 120 patients aged ≥ 55 years, 81 were classified as non-CLT group and the other 39 as CLT-coexisted one (67.5% to 32.5%). The difference in sex proportion between the two groups didn’t reach significance. In addition, no significant difference was shown in lesion size, gross ETE, CLNM or LLNM between the two groups (Table 3).

**Table 3:** Association between CLT and clinicopathological characteristics in patients ≥ 55 years

Charateristics	Total	non-CLT(%)	CLT-coexisted(%)	OR(95%CI)	*P* value
Number of patients	120	81 (67.5)	39 (32.5)		
Sex					
Female	101	65 (80.2)	36 (92.3)	3.0 (0.8–10.8)	0.09
Male	19	16 (19.8)	3 (7.7)		
Small lesion (Dmax ≤ 1 cm)	61	40 (49.4)	21 (53.8)	0.8 (0.4–1.8)	0.29
^#^Mean rank of lesion size		60.97	59.53		0.817
0 < size ≤ 1 cm		40 (49.4)	21 (53.8)		
1 cm < size ≤ 2 cm		18 (22.2)	6 (15.4)		
2 cm < size ≤ 4 cm		20 (24.7)	11 (28.2)		
4 cm < size		3 (3.7)	1 (2.6)		
Extrathyroidal extension	27	20 (24.7)	7 (17.9)	0.7 (0.3–1.7)	0.41
CLNM	24	17 (21.0)	7 (17.9)	0.8 (0.3–2.2)	0.7
LLNM	1	1 (1.2)	0	(0.9–1.0)	0.49
CLNM of small lesions	4	3 (6.3)	1 (4.5)	0.7 (0.1–7.3)	0.63
TNM stage					
I	108	72 (88.9)	36 (92.3)	0.7 (0.2–2.6)	0.56
II-IV	12	9 (11.1)	3 (7.7)		
Multifocality	42	31 (38.3)	11 (28.9)	0.7 (0.3–1.5)	0.32

*CLNM* central lymph node metastasis, *LLNM* lateral lymph node metastasis, *OR* odds ratio, ^#^Mann–Whitney U test used

In patients aged < 55 years, CLT (OR = 1.7, *p* = 0.02) served as the independent risk factor of greater lesion size in multivariate analysis. Female patients were more likely to have small lesions (OR = 0.6, *p* = 0.04). In addition, gross ETE was evidently associated with the larger lesion (OR = 3.3, *p* < 0.001) in multivariate analysis. In terms of macroscopic ETE, CLT coexistence promoted the risk of ETE (OR = 1.4, *p* = 0.06) according to the multivariate analysis, though the trend didn’t reach significance. The greater lesion size also independently predicted ETE presence in return (OR = 3.3, *p* < 0.001). There wasn’t any significant difference in CLNM or LLNM between CLT-coexisted patients and those without, and CLT didn’t cast effect on CLNM (*p* = 0.19). The role of gross ETE took the positive influence on both CLNM (OR = 2.4, *p* < 0.001) and LLNM (OR = 3.1, *p* < 0.001) presence, respectively. Greater lesion size also positively affected CLNM (OR = 1.8, *p* = 0.002) and LLNM (OR = 4.5, *p* < 0.001) (Table 4).

**Table 4:** Association among different clinicopathological characteristics in patients < 55 years

Characteristics	Univariate OR	*P* value	Multivariate OR	*P* value
Greater Size of lesion				
Female	0.7	0.08	**0.6**	**0.04**
Age	**0.9**	**0.006**	**0.9**	**0.02**
CLT	**1.6**	**0.02**	**1.7**	**0.02**
Gross ETE	**3.5**	** < 0.001**	**3.3**	** < 0.001**
Gross ETE				
Female	0.9	0.58	0.9	0.68
Age	1	0.16	1	0.48
CLT	**1.5**	**0.04**	1.4	0.06
Greater Size of lesion	**3.5**	** < 0.001**	**3.3**	** < 0.001**
CLNM				
Female	**0.6**	**0.009**	0.7	0.09
Age	**0.9**	** < 0.001**	0.9	< 0.001
CLT	0.7	0.12	0.7	0.06
Greater Size of lesion	**2.2**	** < 0.001**	**1.8**	**0.002**
Gross ETE	**2.8**	** < 0.001**	**2.4**	** < 0.001**
LLNM				
Female	1.2	0.6	1.6	0.19
Age	0.9	0.22	1	0.59
CLT	0.9	0.67	0.6	0.12
Greater Size of lesion	**5.3**	** < 0.001**	**4.5**	** < 0.001**
Gross ETE	**4.0**	** < 0.001**	**3.1**	** < 0.001**

*ETE* extrathyroidal extension, *CLNM* central lymph node metastasis, *LLNM* lateral lymph node metastasis, *OR* odds ratio

For patients ≥ 55 years, only the greater lesion size worked as an independent factor of gross ETE and CLNM (Table 5).

**Table 5:** Association among different clinicopathological characteristics in patients ≥ 55 years

Characteristics	Univariate OR	*P* value	Multivariate OR	*P* value
Greater Size of lesion				
Female	0.8	0.74	1.3	0.64
Age	1.1	0.04	1.1	0.19
CLT	0.8	0.65	0.9	0.81
Gross ETE	**6.7**	** < 0.001**	**6.0**	**0.001**
Gross ETE				
Female	0.4	0.11	0.5	0.26
Age	1.1	**0.009**	1.1	0.08
CLT	0.7	0.41	0.7	0.56
Greater Size of lesion	**6.7**	** < 0.001**	**5.9**	**0.001**
CLNM				
Female	0.9	0.9	1.1	0.85
Age	1.1	0.29	1	0.79

*ETE* extrathyroidal extension, *CLNM* central lymph node metastasis, *OR* odds ratio

CLT’s role in estimating high-risk recurrent disease was only shown in patients of 35–44 years.

In our whole studied group, only the greater lesion size, instead of CLT, gave rise to high-risk recurrence in the multivariate analysis (OR = 3.5, 95%CI 2.4–5.0, *p* < 0.001). When classified in different age cohorts, the promotion still remained. CLT didn’t significantly affect recurrence stratification in the whole studied group (OR = 1.2, 95%CI 0.8–1.8, *p* = 0.31). According to multivariate analysis in different age cohorts, CLT’s positive effect on high-risk recurrence stratification was shown significant merely in patients aged between 35–44 years (OR = 2.4, 95%CI 1.1–5.4, *p* = 0.02). There didn’t exist evident effect of CLT in other age cohorts in multivariate analysis (Table 6, see Additional file 1).

### Lesion size may positively predict high recurrence risk

Due to the collinearity among variables after taking lesion size subdivision into consideration, the ridge regression takes the role in further analysis. The proper lambda of each age group was calculated using “lm.ridge” in MASS package, which underwent test and being lined in ridge trace (Fig. 1). The “linearRidge” in Ridge package conducted coefficients of independent variables and *P* value, from which “Lesion size” showed significance throughout all age groups. In accordance with Ordinal Logit Regression, CLT was indicated as the significant factor (*p* = *0.01*) to predict high recurrence risks in patients aged between 35 and 44 years old (Table 7, see Additional file 1).

## Discussion

The unambiguous effect of coexisting CLT has always been under debate, due to conflicting data of CLT and risk of malignancy. The contradiction could be attributed to different definition of CLT, contradicted effects of CLT in different age groups, poor pathologic reports of coexistent CLT [35] or variation of statistical approaches. In this study, multivariate analysis was performed to indicate that histopathologically confirmed CLT was independently associated with several aggressive pathologic features in patients < 55 years to some extent, which could suggest in a way that autoimmune thyroiditis harbored unfavorable impacts on overall PTC progress.

It has been indicated in previous abundant data that PTC coexistent with CLT was nearly three times more frequent than non-CLT [36]. Our study has showed that 29.6% of PTC patients had coexistent CLT, which was a consistent frequency with statistics in other reports (0.5–38%) [24, 37–39]. Meanwhile, our study also presented that coexistent CLT was more prevalent in female PTC patients, which was similar to conclusions in other reports [40]. In our entire studied sample, coexistent CLT didn’t show significant association with many aggressive features in malignancy, except that CLT-coexisted group had a greater mean lesion size with a mild trend to have lesions > 1 cm compared with the other group. However, we found that in patients < 55 years, those with coexistent CLT tended to have more extrathyroidal extension and significantly a less frequency of small lesions. While in patients ≥ 55 years, the presence of CLT was associated with none of the clinical or pathologic features.

Our analyzed results indicated that CLT independently predicted macroscopic ETE and larger lesions in patients < 55 years, and this trend was consistent with several previous reports. Babli et al. [31] concluded that the presence of CLT could be in association with unfavorable pathologic features, including ETE in younger patients. Nam et al. [32] observed the larger lesions in CLT-coexisted patients. In contrast, however, CLT played a protective role in cancer development concluded from some other investigations. According to the study from Kim et al. [33], CLT was an independent predictor for low frequency of ETE and CLNM. Our results also suggested an interesting phenomenon that when limited in lesions sized below 1 cm, central lymph node metastasis was less prevalent in CLT-coexisted group, which seemed to contradict CLT’s role in PTC aggressiveness. The controversy may originate from the immunological evidence around CLT and PTC. Considering the fact that papillary microcarcinomas (diameter sized under 1 cm) were a bit common in autoptic observation, CLT was hypothesized to be a timely immune response aimed at impeding PTC progression in the early stage [41].

We observed that the greater lesion size (D_min_ > 1 cm) served as an independent risk factor of gross ETE in patients and was associated with high-risk recurrence stratification in all age cohorts. Besides, ETE and greater lesion size mutually promoted the progression of each other, which had also been supported by other investigations that, according to Kim et al. [33], greater tumor size was independently associated with gross ETE, and vice versa. In the ridge regression performed in different age groups, subdivided greater lesion size consistently deteriorated PTC recurrence risk in the prediction model, which was close to the conclusion reported by Castro et al. [42] that larger lesion size was the excellent predictor of PTC persistence/recurrence. Our observation was also in accordance with studies from Lee et al. [43], that larger tumor size was one of the independent risk factors of gross ETE, which could partly explain the unfavorable prognosis. Apart from the authoritative statement in ATA guideline, gross ETE has been a longtime recognized adverse pathologic predictive factor of worse outcome in many other studies, and the extent of macroscopic ETE appeared to be a potential determinant of PTC recurrence. The macroscopic (gross) extrathyroidal extension has been proposed to be a well-established key variable and of more importance in PTC prognosis stratifications, compared with microscopic ETE [44]. However, it has to be underlined that pathological and clinical features in 2015 ATA risk stratification systems are often diagnosed subjectively and vary in different institutions, so the definite approach to estimating high-risk recurrence still needs further observation so that it can play a part in bettering clinical management.

The results of CLT’s role in initial recurrence risk estimation from the whole studied group didn’t show significance. Interestingly, when divided in different age groups, CLT showed evident predictive value but only in patients aged 35–44 years. No previous investigation has demonstrated CLT’ s role in recurrence risk stratification, especially in different age groups respectively. In that case, our observation could give a hint in regard to subsequent clinical management, of which the focus could be placed on patients in this age group.

In fact, the association between CLT and PTC outcome has remained controversial for over two decades. It is still ambiguous and unexplained partly because of the hesitation in answering whether it is the concurrence or causality between them. One hypothesis elucidated in previous studies pointed out that the lymphocytes migration around tumor lesions (known as “peritumoral infiltration”) was trying to restrict malignancy progression [45]. Kamma et al. [46] observed that lymphocyte infiltration, predominantly around the tumor, was correlated with more aggressive cases. They supposed that surrounding lymphocyte infiltration was followingly induced by the antigen expression on tumor cells, which aroused the necessary but insufficient or even misguided immune attack, with inflammation presence. In support of “inflammation-induced carcinoma”, RET/PTC oncogene has been known to induce a proinflammatory transcriptional program[47]. Human thyrocytes with RET/PTC positivity upregulated a good assortment of proinflammatory cytokines including GM-CSF, M-CSF, IL-1α, IL-1β IL-6,etc. [48, 49]. In addition, inflammation-related enzymes, COX2 for example [50], chemokines like CCL2, CXCL8, CXCL1, CXCL12 and chemokine receptor CXCR4 [51, 52]were also found upregulated from the effect of RET/PTC rearrangement. Other hypotheses were addressed in different ways that the coexisting CLT contributed to cancer progression. Notably, thyroid follicular epithelial cells can show oncocytic changes extensively, exhibiting crowding, irregular contours and large nucleus, which resembled features of papillary carcinoma [53]. According to Giordano et al. [54], the follicular cells in CLT background intensely expressed Fas/FasL due to abundant interleukin-1 beta (IL-1βd), which activated the apoptosis pathway to cause the destruction of normal thyroid tissue, promoting carcinoma growth and malignancy. Despite the abundant reports with regard to the association between CLT and PTC, the exact mechanism is still under debate and in need of further research. Interestingly, the cross-reactivity between peritumor anticancer immunity and autoimmunity was proposed that target-specific antitumor immune response to obscure papillary thyroid microcarcinoma (early stage of PTC) can be misguided to incur destruction of healthy thyroid tissue [55]. With the crucial onset of cross-reactivity, PTC progression was inevitably promoted owing to CLT’s effect in signaling pathway related to apoptosis, increased proliferation rate of tumor cells, etc.[56].

The limitations of our study should be considered that it is a retrospective study, and CLT diagnosis was confirmed on the basis of histopathological examination on thyroid tissue from thyroidectomy, which concealed the real chronological and further causal relationship between PTC and CLT. Meanwhile, the pathological variants of PTC weren’t reported by Pathology Department, which results in the lack of detailed characteristics of tumors in further analysis. Besides, we simply estimate recurrence risks using initial postoperative stratification system, the robust association between CLT, tumor size and recurrence risk needs factual prospective outcomes. The significant results were only applied to patients < 55 years and the predictive value of CLT was only apparent in patients aged in 35–44 years, so the finer investigation towards age specificity and possible mechanisms should be conducted in future study after all.

## Conclusions

Until the controversial association and underlying mechanism between CLT and PTC are clarified, all clinicopathological data and related reasonable speculations should remain on the table for further research. From this study, we conclude that CLT served as an independent risk factor of some aggressive clinical characteristics, including gross extrathyroidal extension and greater lesion size in PTC patients aged below 55 with coexistent CLT. Slightly controversially, for those with maximum diameter under 1 cm, a significant higher proportion of central lymph node metastasis was presented without evidence of CLT. The contradictory phenomenon may be attributed to the unclear chronological order and causality of the occurrence of CLT and PTC.

Moreover, in the light of authoritative stratification from ATA guideline combined with our results, lesion size was associated with high-risk recurrence prediction in all age groups and CLT-coexisted PTC patients aged in 35–44 years were estimated more likely to undergo high-risk recurrence. Larger size hints the trend to recurrence in PTC patients, so the adequate therapeutic planning as well as close follow-up, including attention paid on Tg concentration and imaging evidence after surgery, should be in force. As for predicting high-risk recurrence from coexistent CLT in patients aged between 35 and 44 years old, the expectation of further prospective study is placed in order to unearth the true nature in this phenomenon.

## Supplementary Information

**Additional file 1.**

**Additional file 2.**

## Data Availability

The data analyzed that support the findings of this study are available from the corresponding author upon reasonable request.

## Abbreviations

PTC: Papillary thyroid cancer
CLT: Chronic lymphocytic thyroiditis
ATA: American Thyroid Association
CLNM: Central lymph node metastasis
LLNM: Lateral lymph node metastasis
ETE: Extrathyroidal extension
